# Exploring Determinants of COVID-19 Vaccine Acceptance, Uptake, and Hesitancy in the Pediatric Population: A Study of Parents and Caregivers in Saudi Arabia during the Initial Vaccination Phase

**DOI:** 10.3390/healthcare11070972

**Published:** 2023-03-29

**Authors:** Abdullah N. Alhuzaimi, Abdullah A. Alrasheed, Ayman Al-Eyadhy, Fadi Aljamaan, Khalid Alhasan, Mohammed A. Batais, Amr Jamal, Fatimah S. Alshahrani, Shuliweeh Alenezi, Ali Alhaboob, Fahad AlZamil, Yaser Y. Bashumeel, Ahmad M. Banaeem, Abdulrahman Aldawood, Rabih Halwani, Mazin Barry, Jaffar A. Al-Tawfiq, Mohamad-Hani Temsah

**Affiliations:** 1 College of Medicine, King Saud University, Riyadh 11362, Saudi Arabiamtemsah@ksu.edu.sa (M.-H.T.); 2 Division of Pediatric Cardiology, Cardiac Science Department, College of Medicine, King Saud University Medical City, Riyadh 11362, Saudi Arabia; 3 Pediatric Cardiology Section, Heart Center, King Faisal Specialist Hospital & Research Center, Riyadh 11211, Saudi Arabia; 4 Department of Family and Community Medicine, King Saud University Medical City, Riyadh 11362, Saudi Arabia; 5 Pediatric Department, King Saud University Medical City, Riyadh 11362, Saudi Arabia; 6 Critical Care Department, King Saud University Medical City, Riyadh 11362, Saudi Arabia; 7 Solid Organ Transplant Center of Excellence, King Faisal Specialist Hospital and Research Center, Riyadh 11564, Saudi Arabia; 8 Family Medicine Center, King Saud University Medical City, Riyadh 11362, Saudi Arabia; 9 Evidence-Based Health Care & Knowledge Translation Research Chair, Family & Community Medicine Department, College of Medicine, King Saud University, Riyadh 11362, Saudi Arabia; 10 Division of Infectious Diseases, Department of Internal Medicine, King Saud University Medical City, Riyadh 11362, Saudi Arabia; 11 Department of Psychiatry, King Saud University, Riyadh 11362, Saudi Arabia; 12 College of Medicine, Sulaiman Al Rajhi University, Al-Bukayriah 51941, Saudi Arabia; 13 College of Medicine, Imam Mohammed Ibn Saud Islamic University, Riyadh 11432, Saudi Arabia; 14 Department of Clinical Sciences, College of Medicine, University of Sharjah, Sharjah 27272, United Arab Emirates; 15 Research Institute for Medical and Health Sciences, University of Sharjah, Sharjah 27272, United Arab Emirates; 16 Department of Internal Medicine, College of Medicine, University of Ottawa, Ottawa, ON K1H 8M5, Canada; 17 Specialty Internal Medicine and Quality Department, Johns Hopkins Aramco Healthcare, Dhahran 34465, Saudi Arabia; 18 Infectious Disease Division, Department of Medicine, Indiana University School of Medicine, Indianapolis, IN 46202, USA; 19 Infectious Disease Division, Department of Medicine, Johns Hopkins University School of Medicine, Baltimore, MD 21218, USA; 20 Prince Abdullah bin Khaled Coeliac Disease Research Chair, King Saud University, Riyadh 11362, Saudi Arabia

**Keywords:** COVID-19 vaccine uptake in children, parental vaccine hesitancy, Vaccine Hesitancy Scale (VHS), COVID-19 vaccine and routine childhood vaccinations

## Abstract

Objectives: This study aims to assess COVID-19 vaccine acceptance, uptake, and hesitancy among parents and caregivers of children in Saudi Arabia during the initial rollout of pediatric COVID-19 vaccination. Methods: An electronic survey was used to collect data from participants who visited a COVID-19 vaccine center. The survey included demographic data, COVID-19 vaccine status among participants and their children, and reasons for vaccine acceptance or rejection. The Vaccine Hesitancy Scale (VHS) tool was also employed to assess vaccine hesitancy and attitudes toward the COVID-19 vaccine and routine childhood vaccination. Multivariate binary regression analysis was used to identify predictors of actual COVID-19 vaccine uptake among children. Results: Of the 873 respondents included in the analysis, 61.5% were parents and 38.5% were other caregivers. Of the participants, 96.9% had received the COVID-19 vaccine. Six hundred and ninety-four participants accepted the vaccine for their children, with the main reasons being an endorsement by the Saudi Ministry of Health (60%) and the importance of going back to school (55%). One hundred and seventy-nine participants would not vaccinate their children, with the most common reasons being fear of adverse effects (49%) and inadequate data about vaccine safety (48%). Factors such as age, COVID-19 vaccination status, self-rated family commitment level, attitudes toward routine children’s vaccines, and participants’ generalized anxiety disorder (GAD7) score did not significantly correlate with children’s COVID-19 vaccination status. Parents were less likely to vaccinate their children compared to other caregivers, and participants with a higher socioeconomic status were more likely to vaccinate their children. Conclusion: Vaccine acceptance and uptake were high during the initial pediatric COVID-19 vaccination rollout in Saudi Arabia. Still, the ongoing endorsement of the Ministry of Health and healthcare authorities should continue to advocate for better vaccine uptake in children.

## 1. Introduction

Since the emergence of the severe acute respiratory syndrome coronavirus 2 (SARS-CoV-2), there had been limited therapeutics for the prevention and treatment of the disease and the management of such patients is complex. Thus, it was important to have effective vaccines for the prevention of severe disease with the hope to prevent transmission. COVID-19 vaccines were initially rolled out for adults in late 2020 and, subsequently, the US Centers for Disease Control and Prevention (CDC) approved the use of mRNA COVID-19 vaccines such as BNT162b2 for children 12–15 years of age in May 2021, followed by the approval for children aged 5–11 years in November 2021, and it was finally approved for children aged between 6 months to 4 years old in February 2022 [1,2,3].

School-age children represent a high proportion of coronavirus disease 2019 (COVID-19) cases [4], therefore they may play an important role in the transmission of the virus [5,6]. Vaccination of this age group ahead of emerging mutants and waves of the disease will offer protection from serious illness and hospitalization and will augment community herd immunity [7].

Results of an ongoing phase 1–2–3 clinical study in healthy persons 12 years of age or older support the safety, immunogenicity, and efficacy of a two-dose series of 30 μg BNT162b2 injections administered 21 days apart [1,8]. BNT162b2 has been shown to have an acceptable safety profile characterized by transient mild-to-moderate injection-site pain, fatigue, and headache, it was shown to be more immunogenic among children aged 12 to 15 years old than among young adults, and was 95 to 100% efficacious in preventing COVID-19 7 days to approximately 2 months after the second dose [9]. Vaccine efficacy against COVID-19 from 7 days to 6 months after the second dose was 91%, with a similar estimated real-world effectiveness from 8 to 28 days after the second dose [10]. However, based on the Omicron variant, the vaccine efficacy after 7 days of vaccination was 48% for three doses and 69% for four doses [11].

Despite documented vaccine safety and efficacy to reduce mortality and serious disease, there is ongoing vaccine hesitancy among the public around the globe. Lazarus et al. documented an overall vaccine hesitancy of 24% among the population in 23 different countries surveyed in June 2021 [12]. The same study showed a higher vaccine hesitancy among parents to vaccinate their children, with a vaccine hesitancy of 5–64.5% and an overall global average of 32% [12].

In the Kingdom of Saudi Arabia, the Saudi Food and Drug Authority (SFDA) approved the COVID-19 vaccine for adults on 10 December 2020, and for children 12–18 years of age on 27 June 2021 and for children 5–11 years of age in November 2021. An initial survey early 2020 in Saudi Arabia showed that 64.7% of adult respondents were willing to receive a COVID-19 vaccine once introduced [13]. A national survey among parents carried out in Saudi Arabia in 2021 showed 52.4% of parents were either undecided or refused to vaccinate their children (12–18 years) [14]. It also showed a greater parental vaccine hesitancy score towards the COVID-19 vaccine compared to routine childhood vaccination. Another study carried out in Saudi Arabia between 31 December 2021 and 7 January 2022 showed only 32.1% of parents were willing to vaccinate children (5–11 years) and half already vaccinated their children aged 12–18 years, in addition to 31% who were planning to vaccinate them [15].

Therefore, there is still major hesitancy among parents to vaccinate their children in Saudi Arabia [16]. In addition, there are limited data about the reasons for accepting or rejecting the COVID-19 vaccine among parents and caregivers for children aged 12–18 years. In this study, we measure vaccine acceptance as represented by vaccine uptake, explore vaccine hesitancy using the Vaccine Hesitancy Scale, and review the predictors of childhood COVID-19 vaccine acceptance or rejection as seen by parents and caregivers of children in Saudi Arabia. We aim to enhance the global literature on vaccine hesitancy and contributing factors in order for public health practitioners to develop effective communication plans and campaigns for such vaccination.

## 2. Methods

A cross-sectional survey on visitors of the King Saud University vaccination center in Riyadh, Saudi Arabia during the initial phase of the pediatric COVID-19 vaccination rollout between 15 July 2021 to 25 September 2021 was carried out. Parents and caregivers who agreed to participate in the survey were given an electronic web-based survey (using the SurveyMonkey platform) using a QR code.

The COVID-19 vaccine centers were open to all the public to receive their vaccination based on bookings from the Ministry of Health through the COVID-19 Vaccine Booking Service [17].

The survey was formulated based on a review of the related literature including our group’s previous work [14,18,19,20,21]. Caregiver was defined as a family member who regularly looks after the child.

The survey consists of 4 sections: the first section captured the demographics of the parents and caregivers; the second section explored participants’ COVID-19 perceptions and uptake of the COVID-19 vaccine among the participants and their children, families’ commitment level to the precautionary measures against COVID-19, the reasons for avoiding or accepting the COVID-19 vaccine in children, and sources of information utilized about COVID-19 vaccines.

The third section consisted of GAD7 scale items to assess the level of generalized anxiety among participants; the fourth section was formulated to assess vaccine hesitancy [22]. We employed a modified version of the WHO’s Strategic Advisory Group of Experts on Immunization Vaccine Hesitancy Scale (VHS) to assess COVID-19 vaccine hesitancy as compared to routine childhood vaccination, similar to our previous publication [14]. The WHO VHS tool was initially developed in 2015 by the SAGE Working Group on Vaccine Hesitancy and built upon extensive global pilot data of indicators for vaccine hesitancy [23].

In a similar fashion to Kempe et al. [19], we altered the Likert scale into a 4-point scale, excluding the “neutral or not sure” option, as recent evidence suggests that a 4-point scale without the choice of neutrality reduces the potential for social conformity. The wording was slightly revised from the original VHS to tackle both the COVID-19 vaccine and childhood vaccination to create a point of comparison. In addition, all items except “New vaccines carry more risks than older vaccines” were asked twice: once concerning the COVID-19 vaccine and once in general, to create a point of comparison for our analysis.

The survey invitation clarified the inclusion criteria, being an adult caregiver in Saudi Arabia, and voluntary participation. Additionally, the survey stressed the privacy of data by not seeking any individual identifiers. This survey was already translated and back-translated into the Arabic language and was piloted among 52 parents prior to being used in our previous publication and, overall, all items were reliable and were understood equally reliably by people, with Cronbach’s alpha = 0.87 [14].

The non-linear (categorical) factor analysis was applied to people’s measured socioeconomic characteristics (namely: the household’s size, marital status, household monthly income, and employment). The resulting factor analysis results showed that these four indicators had loaded significantly and saliently (well above 0.42 for each of them) to a single latent factor that was named as socioeconomic index (SES-I). People with a higher socioeconomic index score had a significantly greater family size, more household income, tended to be married, and were employed) and, vice versa, people with lower socioeconomic index were single, unemployed, with smaller family size, and had lower monthly household income. The estimated SES-I score was estimated through the R program. The resulting estimate of the SES-I had a mean equal to zero and a standard deviation equal to one, and this SES index was used as an independent predictor in the multivariable analysis models used in the study.

Cronbach’s alpha test of internal consistency showed that the Vaccine Hesitancy Scale was reliable when applied to measure both parents’ attitudes toward both routine and COVID-19 vaccines with Cronbach’s alpha = 0.75 for children’s COVID-19 vaccination hesitancy and Cronbach’s alpha = 0.79 for routine children’s vaccination.

In addition, the categorical Cronbach’s alpha yielded from the categorical factor analysis of the respondents’ socioeconomic factors showed acceptable internal consistency, Cronbach’s alpha = 0.70.

### 2.1. Sample Size

For the sample size estimation, using the sample size calculator for proportion, and assuming that 50% of the sample will demonstrate sufficient vaccine uptake, the minimum desired sample size that would be required to detect a true proportion of participants, with a confidence level of 95% and a margin of error of 5%, was estimated to be equal to 384 subjects.

### 2.2. Statistical Analyses

Continuous variables were described by the mean and standard deviation, and the frequency and percentages were used to describe the categorical variables. The multiple response dichotomy analysis was applied to the measured variables with more than one option (“tick all that apply” questions). The mean parental attitude (VHS) score toward vaccines was computed by averaging the eight items comprising the scale after reverse ordering the negatively worded statements so that a higher score denoted a better attitude toward vaccines, then attitude VHS scores were dichotomized into low versus high hesitance toward vaccines based on a cut off value (=3 points); thus, people with a mean attitude score lower than 3 VHS attitude points were considered to have high hesitance (negative attitude) and people with a ≥ 3 VHS score were considered to have low hesitance (positive attitude) toward vaccines. The paired samples *t*-test was used to compare people’s attitudes toward routine and COVID-19 vaccines.

The multivariate binary regression analysis was used to assess the statistical significance of the predictors of people’s odds of actual uptake of the COVID-19 vaccine for their children. The association between the odds of vaccine uptake with their sociodemographic and other relevant predictor variables was expressed as an odds ratio with associated 95% confidence interval. The alpha significance level was considered at the 0.050 level. The SPSS IBM statistical analysis program, version 21, was used for the data analysis.

## 3. Results

### 3.1. Participants’ Sociodemographic Characteristics

The sociodemographic characteristics of the 873 respondents who were included in the analysis are shown in Table 1. Most caregivers were parents, at 61.5%. The majority of respondents (38.2%) were young, at 18–24 years old. Additionally, 62.5% of them had a child or sibling who is 12–18 years of age.

About one-third the respondents (36.5%) were either unemployed or were housewives, another 11.7% of them worked in freelance jobs, and 11.5% of them were healthcare workers, but most of them (40.3%) were employed in either private or public sectors.

**Figure 1:**
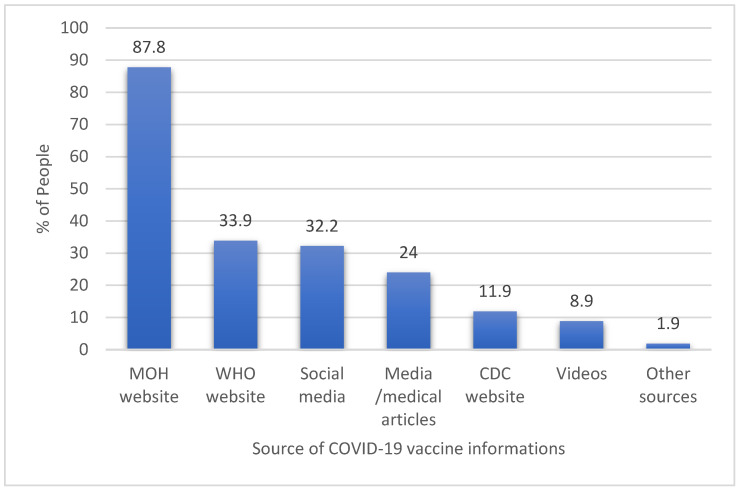
Caregivers’ sources of children’s COVID-19 vaccine information.

Most of the participants (87.8%) used the Saudi Ministry of Health (MOH) website for information and updates on COVID-19 vaccines in children, followed by the WHO’s online resources. The parents’ other sources of information about COVID-19 are shown in Figure 1.

### 3.2. Participants’ COVID-19 Perceptions and Vaccine Uptake

Most surveyed participants (96.9%) had already received the COVID-19 vaccinations. In relation to whether their children/sibling aged ≥12 years old had taken the COVID-19 vaccines, 48.2% had already received a COVID-19 vaccine, 31.3% were planning to vaccinate their children, and the rest either had not received the vaccine or were not sure. Almost one-third of respondents had and a family member with SARS-CoV-2. The mean perceived commitment to COVID-19 precautionary measures was self-rated as 4.10/5 points, SD = 1.2 points. Further details are shown in Table 2 with 62.1% thinking they were highly committed to precautionary measures. The overall generalized anxiety (GAD7) score showed a relatively low level of anxiety with a mean score of 5.01/21 points with only 8.1% having a moderate level of anxiety and 6.2% having high anxiety levels.

### 3.3. Reasons Affecting the Acceptance or Rejection of COVID-19 Vaccine in Children

**Figure 2:**
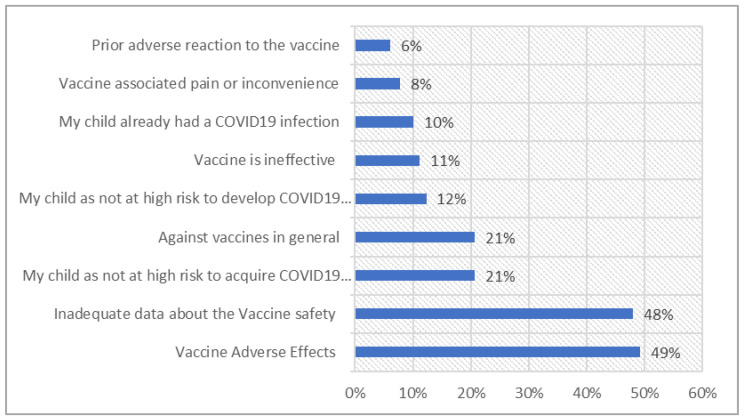
Reasons for parental avoidance of the COVID-19 vaccine in children (n = 179).

People who stated that their children aged > 12 did not receive COVID-19 vaccines (n = 179) were asked about the reasons for vaccine rejection (Figure 2).

**Figure 3:**
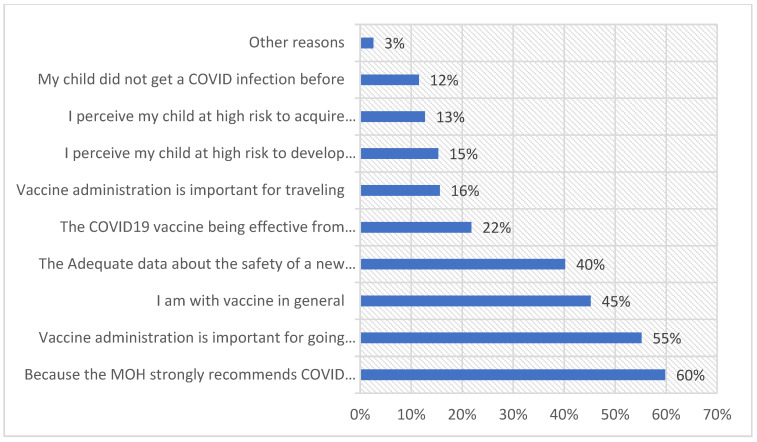
Reasons for vaccinating children against COVID-19 (n = 694).

Almost half of them were concerned about potential side effects on their child or believed that there is inadequate information on the safety of the COVID-19 vaccines. Another 20.7% of them suggested that they are against the vaccine in general and/or believed their child is not at high risk for acquiring the disease. On the other hand, participants who vaccinated their children or planned to vaccinate them were asked for the motives for vaccinating their children against COVID-19 (Figure 3).

Almost 60% stated they vaccinated their children because of the MOH’s strong recommendation, 55.2% thought the vaccines were necessary for children to be able to go back to school, and almost 45.2% stated they agree with vaccines in general. In addition, 40.2% of them believed that there were sufficient data about the vaccine’s safety. Other reasons are shown in Figure 3.

### 3.4. Vaccine Hesitancy Scale (VHS) for COVID-19 Vaccine vs. Childhood Vaccines

The participants’ attitude toward the COVID-19 vaccine versus routine childhood vaccines was measured using a modified VHS. The level of agreement was measured using eight different statements for both the COVID-19 vaccine and routine childhood vaccines with a scale from 1–4 (Table 3).

The respondents’ overall attitude toward both vaccine types was measured via computing the average of the indicators comprising each of the scales so that greater score indicated a more positive attitude. The analysis showed that respondents’ collective mean attitude toward routine childhood vaccines was significantly better than their mean score toward the COVID-19 vaccine for children (3.08 ± 0.48 versus 2.90 ± 0.57) with a mean difference of 0.19 (95% C.I 0.16:0.214, *p*-value < 0.00). A bivariate Pearson’s correlation test showed that participants’ attitudes toward routine childhood and COVID-19 vaccines were correlated positively, r = 0.720, *p*-value < 0.010.

In addition, the respondents’ attitudes toward vaccines were dichotomized based on VHS score into positive attitude (≥3 VHS) and negative attitude (VHS < 3) for both routine childhood vaccines and the COVID-19 vaccine. It was found that 60.1% have a positive attitude (i.e., agreement) toward routine children’s vaccinations while 48.1% were found to have a positive attitude toward the COVID-19 vaccinations.

### 3.5. Multivariate Analysis of Actual Uptake of COVID-19 Vaccine in Children

The predictors of the actual uptake of COVID-19 vaccine in children aged >12 years were analyzed using a multivariate binary logistic regression showing an adjusted odds ratio for various participant characteristics (Table 4). The analysis showed that parents of children were found to be significantly less likely (67.5% times less) to have children vaccinated against COVID-19 compared to other respondents, *p*-value < 0.001. Respondents’ age groups did not correlate significantly with the children’s vaccination uptake, nor did the participants’ COVID-19 vaccination status or their self-rated family commitment toward COVID-19 precautionary behaviors.

The households’ socioeconomic index (SES score) correlated significantly and positively with the children’s uptake of the COVID-19 vaccine. For each additional standard point in the household’s socioeconomic index, the children’s odds of COVID-19 vaccine uptake tended to rise by a factor equal to 77.1% on average, *p*-value < 0.001. However, participants with a high school educational level were found to be significantly less likely (78.9% times less) to vaccinate their child against COVID-19 compared to those with middle school education on average, *p*-value = 0.004. Those with a university degree were found to be significantly less likely (83.8% times less) to vaccinate their child against COVID-19 compared to those with a middle school or lower educational level on average, *p*-value = 0.001.

Having a child with physical or psychological illness did not correlate significantly with children’s COVID-19 uptake. Although the odds of COVID-19 vaccine uptake were 1.3:1 among children of participants who have a positive attitude toward the COVID-19 vaccine, the relationship was not statistically significant. Neither the generalized anxiety score nor a positive attitude toward children’s routine vaccines correlated significantly with COVID-19 vaccination uptake.

## 4. Discussion

The introduction of the COVID-19 vaccine was one of the most important factors to contain the pandemic. However, the introduction of the vaccine opened the gate for increased hesitancy and vaccine refusal around the globe. In a systematic review of the acceptance of the COVID-19 vaccine among adults, significant variations in vaccine acceptance among different countries were found [24]. Moreover, a recent systematic review reported that the global overall pooled acceptance rate of the COVID-19 vaccine was 64.9% [25]. In another systematic review of childhood COVID-19 vaccine hesitancy, higher vaccine hesitancy rates were documented in the USA and reached up to 86.1% and >85% in Saudi Arabia and Turkey [26]. Vaccine hesitancy was observed in individuals with a history of poor influenza vaccination uptake and those who believe in conspiracy theories linking vaccination to infertility, along with exposure to misinformation about COVID-19 vaccines on social media [24].

In our study, 96.9% of the surveyed participants had already received a COVID-19 vaccine and 48.2% of their children aged > 12 years of age had received the vaccine. In addition, 31.3% were planning to vaccinate their children. The vaccination status significantly improved over time in the Kingdom of Saudi Arabia. The improvement is related to multiple factors including increased awareness and staged implementation of the vaccination policy in the country [27].

In an earlier study, 47.6% intended to vaccinate their children with a COVID-19 vaccine, a finding that is lower than the current overall rate of 79.5% who had already received the vaccine or planned to have their children vaccinated [14]. Although vaccination for this age group has generally been recommended, improvement in the rate of vaccination indicates more acceptance of the vaccine in this age group. It was noted that parents had generally more positive attitudes toward routine childhood vaccination than childhood COVID-19 vaccine [14]. Caregivers were more likely to accept COVID-19 vaccination compared to parents, which could be because caregivers already had parental permission before they attended the center or had knowledge about the significance of receiving the vaccine to protect from COVID-19 infection.

The main reason for the refusal of childhood COVID-19 vaccination was the concern about the risk and possible adverse reactions. The cited concern is consistent with previous studies of COVID-19 vaccine uptake in children [28,29,30].

In the present study, parents of children and the participants with high school or university level education were found to be significantly less likely to have children vaccinated against COVID-19. Byrne et al. conducted a cross-sectional survey to determine parental acceptance of vaccines against COVID-19 for their school-aged children and reported no significant association of parental education with vaccination against COVID-19 for their children [30]. However, they reported that parents with high school education or less were more likely to trust medical professionals than those parents with higher education. This statement supports the findings of the present study in terms of parental education and acceptance of vaccination for their children. In contrast, Padhi et al. conducted an online cross-sectional national survey in India to assess parents’ perceptions and intentions to vaccinate their children against COVID-19, reporting greater likelihood of child vaccination among parents having a bachelor’s degree or higher education (OR: 1.98, 95% CI: 1.15–3.51) [31]. Similarly, Aedh conducted a cross-sectional survey in Najran, Saudi Arabia, reporting that parents with graduate or postgraduate education were more inclined to accept or approve of vaccination against COVID-19 for their children [32]. The reason behind these findings may be the use of online social media platforms or the fact that parents having higher education levels are four times more concerned about vaccine safety than those with lower education levels [33]. In addition, this might be the result of the poor role played by community institutions in regard to increasing public awareness about the significance of taking the COVID-19 vaccine and the significant distrust in government and health organizations [34].

The family households’ SES score correlated significantly and positively with children’s uptake of the vaccine against COVID-19. Similar findings have been reported by Aedh et al. that parents who earned more money had greater intention to vaccinate their children [32]. Similarly, although not statistically significant, Alfieri et al. conducted an online cross-sectional survey in Illinois, USA and reported higher vaccine hesitancy among lower income groups [35]. Similar findings have been reported in England that lower income households were more likely to oppose vaccination against COVID-19 [36]. In addition, Joshi et al. have reported that acceptance of vaccination is higher among those with high income [37]. This shows that a family’s income is an important factor for the acceptance of vaccination against COVID-19 for children.

In the present study, age, COVID-19 vaccination status, self-rated family commitment level, positive attitudes toward routine children’s vaccines and COVID-19 vaccines, and generalized anxiety disorder score of participants did not correlate significantly with the children’s true COVID-19 vaccination status. Like the findings of the present study, Padhi et al. and Bell et al. reported no significant association of parents’ age and acceptance of children’s COVID-19 vaccination [31,36]. Byrne et al. observed significantly higher odds of vaccinating children among parents aged 40 or above [30]. This study reported that parents who had received a COVID-19 vaccine had greater odds for vaccinating their children (2.1:1) but the relation was statistically not significant in multivariate analysis (*p* = 0.09). Adeh also reported higher willingness among parents who received the COVID-19 vaccination for vaccination of their children as compared to those who did not receive vaccination [32]. A positive attitude toward the COVID-19 vaccine as measured by the VHS did not correlate with vaccine uptake in this study. The higher uptake despite vaccine hesitance might be due to the effect of strong recommendation by local authorities for the COVID-19 vaccine for children to allow safe return to schools. Other reasons are shown in Figure 3.

Additionally, the present study has reported that having a child with physical/psychological illness does not significantly affect children’s COVID-19 vaccination status. This finding can be studied further in future studies. To increase vaccine uptake in children, strategies could include increasing awareness through various channels, collaborating with multidisciplinary healthcare providers and school health professionals, involving community leaders and organizations, simplifying the vaccination process, creating a positive and supportive environment, monitoring vaccine acceptance rates, and adjusting strategies as needed [38,39].

Additionally, utilizing new technology, such as artificial intelligence (AI) chatbots (such as ChatGPT) can also provide additional information to parents and caregivers about the benefits and safety of the COVID-19 vaccine for their children [40]. ChatGPT can address common concerns and misconceptions related to vaccines and provide personalized recommendations based on individual circumstances [41]. This technology can be integrated into healthcare systems, websites, and mobile applications to reach a wider audience and provide support and education on vaccine uptake, but this needs further research [42].

This study’s international contribution to the literature arises from its investigation of the factors influencing parental acceptance and hesitancy toward COVID-19 vaccination for their children. This finding contributes valuable insights to the expanding evidence base on vaccine hesitancy worldwide by emphasizing the role of educational level, family income, and vaccine safety concerns [43]. These findings can help inform public health strategies and communication initiatives that target specific groups and tackle their issues, resulting in higher vaccine acceptance rates and, ultimately, better pandemic control.

Furthermore, the study’s objectives are to investigate parents’ and caregivers’ attitudes, beliefs, and intentions regarding childhood COVID-19 vaccination in the Kingdom of Saudi Arabia. The study provides critical information for policymakers to develop culturally relevant and directed measures to tackle vaccine hesitancy and enhance vaccine uptake by investigating the relationship between socio-demographic factors, parental education, and vaccine acceptance [44,45].

Finally, the study adds to the public health literature by highlighting the significance of understanding and addressing vaccine hesitancy, especially in light of the ongoing pandemic. This research offers helpful insights for health officials and professionals to develop evidence-based strategies that adequately address concerns and boost vaccine acceptance, inevitably assisting in the worldwide fight against COVID-19 [24].

### Study Strengths and Limitations

Our study demonstrated several strengths and limitations. While our study is one of the first studies to address parents’ reasons to give or not to give their children the COVID-19 vaccine, it has a few limitations. The study was conducted using a convenience sampling method which might have introduced selection bias that may have affected the results. Still, convenience sampling methods are acceptable during healthcare emergencies [46]. Our exploratory research at the vaccine center had an additional rationale during the Delta variant wave. While the adults’ vaccinations were mandatory, children’s vaccination was initially optional. Therefore, gaining rapid insights into the caregivers’ reasons for accepting or rejecting their children’s vaccines could be essential to enlighten policymakers.

In addition, given the survey is based on closed-ended questions, could not address all possible factors that contributed to parents’ hesitancy toward the childhood COVID-19 vaccine, but was mainly limited to factors of interest. Future research could incorporate more parents in other settings to evaluate the external validity of our research. In addition, national surveys could be further conducted to assess the changing patterns of acceptance or hesitancy toward vaccines, on a longitudinal, surveillance basis that could better inform policymakers of adjustments to improve vaccine uptake.

The online survey has limitations including potential bias related to self-report questionnaires, limited accessibility and use of the internet which may have led to sample bias, and the sample may not be representative of the broader population. These limitations should be considered when interpreting the results.

## 5. Conclusions

There was high COVID-19 vaccine acceptance among parents and caregivers in Saudi Arabia in the initial rollout among the pediatric population. There still a degree of vaccine hesitancy toward the COVID-19 vaccine, especially among parents and participants with a higher educational level. In addition, a higher socioeconomic index score was associated with higher acceptance of children’s vaccination against COVID-19. Further studies at a large scale are required to support these findings of the present study.

## Figures and Tables

**Table 1:** Parental sociodemographic characteristics. N = 873.

Relationship to the Child	Frequency	Percentage
Mother	352	40.3
Father	185	21.2
Other relative	336	38.5
**Age group**		
18–24 years	334	38.3
25–34 years	145	16.6
35–44 years	200	22.9
45–54 years	151	17.3
55 years or higher	43	4.9
**Marital status**		
Married, divorced, or widowed	537	61.5
Single	336	38.5
**Household size (members), mean (SD)**		5.85 (2.42)
**Do you have a child/sibling whose age is 12–18 years**		
No	327	37.5
Yes	546	62.5
**Was this child (aged 12–18 years) ever diagnosed with an physical or psychological illness?**		
No	847	97
Yes	26	3
**Educational level**		
Middle school or less	26	3
High school	214	24.5
University	633	72.5
**Households’ monthly income**		
Prefer not to answer	213	24.4
Less than SR 5000 (USD 1333)	70	8
SR 5000–10,000 (USD 1333–2666)	162	18.6
More than SR 10,000 (USD 2666)	428	49
**Employment**		
Unemployed	319	36.5
Freelance job	102	11.7
Healthcare worker	100	11.5
Employed	352	40.3

**Table 2:** Participants’ COVID-19 perceptions and uptake of the vaccine.

	Frequency	Percentage
**Did you take the COVID-19 vaccine?**		
Yes	846	96.9
No	27	3.1
**Did any of your children/siblings (aged ≥ 12 years) receive the COVID-19 vaccine?**		
Yes: my child already received the COVID-19 vaccine	421	48.2
I plan to vaccinate my child	273	31.3
No	100	11.5
Not sure	79	9
**Have any direct family members been affected by COVID-19?**		
No	549	62.9
Yes	324	37.1
Describe your family’s commitment to the precautionary measures against the COVID-19 virus, mean (SD) *		4.10 (1.20)
**Describe your family’s commitment to the precautionary measures against the COVID-19 virus**		
Low commitment	65	7.4
Moderate commitment	266	30.5
High commitment	542	62.1
**Parents’ generalized anxiety (GAD7) score, mean (SD)**		5.01 (5.04)
**Parents’ generalized anxiety (GAD7) classification**		
Very low: <5 points	475	54.4
Mild: 5–10 points	273	31.3
Moderate: 11–14 points	71	8.1
High: >15 points	54	6.2

* Likert-like scale graded from 1 = rarely committed to 5 = very highly committed.

**Table 3:** Parental attitudes toward children’s COVID-19 vaccine.

Attitudes Toward Routine Childhood Vaccines	Mean *	SD
Childhood routine vaccines are essential for my child’s/children’s health	3.39	0.7
2. Childhood vaccines are effective	3.3	0.65
3. It is important for the health of others in the community that my child/children be vaccinated	3.23	0.73
4. The information I acquire about vaccines from vaccine programs is credible	3.06	0.66
5. My child/children getting childhood vaccines is a good way to protect them from disease	3.25	0.66
6. Generally, I follow what my doctor or health care provider suggests about childhood vaccines	3.3	0.59
7. I am anxious about serious adverse effects of routine children’s vaccines	2.54	0.85
8. My child/children do not need vaccines for diseases that are not common anymore	2.29	0.86
**Attitudes Toward COVID-19 Vaccines**		
COVID-19 vaccine is essential for my child’s/children’s health	3.05	0.086
2. COVID-19 vaccine is effective	3.05	0.78
3. It is important for the health of others in the community that my child/children be COVID-19 vaccinated	3.13	0.8
4. New vaccines (like COVID-19?) bear more risks than older vaccines	2.52	0.84
5. The information I acquire about the COVID-19 vaccine from its vaccine program is credible	3.04	0.69
6. My child/children getting COVID-19 vaccine is a good way to protect them from the virus	3.08	0.78
7. Generally, I follow what my doctor or health care provider suggests about COVID-19 vaccine for my child/ren	3.19	0.69
8. I am anxious about the serious adverse effects of the COVID-19 vaccine	2.82	0.87

* 1–4 Likert-like scale coded as follows (1 = strongly disagree, 2 = disagree 3 = agree, and 4 = strongly agree).

**Table 4:** Multivariate binary logistic regression of actual uptake of children’s COVID-19 vaccine.

	Multivariate Adjusted Odds Ratio	95% CI for OR		*p*-Value
		Lower	Upper	
Parent = Yes	0.325	0.195	0.540	<0.001
Age group	1.186	0.999	1.408	0.052
COVID-19-vaccinated persons	2.124	0.884	5.102	0.092
Family level of COVID-19 precautionary commitment mean score	1.233	0.985	1.544	0.068
Household socioeconomic status index (SES-I) score	1.771	1.417	2.212	<0.001
Educational level = High school	0.211	0.074	0.608	0.004
Educational level = University degree	0.162	0.058	0.454	0.001
Having a child/sibling who is mentally/physically disabled or diseased	0.439	0.185	1.041	0.062
Positive attitude toward COVID-19 vaccine	1.301	0.914	1.851	0.144
Positive attitude toward routine children’s vaccines	1.150	0.803	1.646	0.446
Generalized anxiety (GAD7) score	1.007	0.979	1.036	0.612
Constant	1.545			0.569

DV = actual vaccination of children aged >12 years (No/Yes).

## Data Availability

Data will be made available upon reasonable request directed to mtemsah@ksu.edu.sa.

## Abbreviations

CDC: Centers for Disease Control and Prevention; COVID-19: Coronavirus Disease 2019; GAD7: Generalized Anxiety Disorder Assessment; MOH: Ministry of Health; SARS-CoV-2: Severe Acute Respiratory Syndrome Coronavirus 2; VHS: Vaccine Hesitancy Scale; WHO: World Health Organization.
